# Molecular mechanisms of multi-omic regulation in breast cancer

**DOI:** 10.3389/fonc.2023.1148861

**Published:** 2023-07-25

**Authors:** Soledad Ochoa, Enrique Hernández-Lemus

**Affiliations:** ^1^ Computational Genomics Division, National Institute of Genomic Medicine, Mexico City, Mexico; ^2^ Department of Obstetrics and Gynecology, Cedars-Sinai Medical Center, Los Angeles, CA, United States; ^3^ Center for Complexity Sciences, Universidad Nacional Autónoma de México, Mexico City, Mexico

**Keywords:** multiomics, breast cancer, DNA methylation, epigenomic regulation, network biology

## Abstract

Breast cancer is a complex disease that is influenced by the concurrent influence of multiple genetic and environmental factors. Recent advances in genomics and other high throughput biomolecular techniques (-omics) have provided numerous insights into the molecular mechanisms underlying breast cancer development and progression. A number of these mechanisms involve multiple layers of regulation. In this review, we summarize the current knowledge on the role of multiple omics in the regulation of breast cancer, including the effects of DNA methylation, non-coding RNA, and other epigenomic changes. We comment on how integrating such diverse mechanisms is envisioned as key to a more comprehensive understanding of breast carcinogenesis and cancer biology with relevance to prognostics, diagnostics and therapeutics. We also discuss the potential clinical implications of these findings and highlight areas for future research. Overall, our understanding of the molecular mechanisms of multi-omic regulation in breast cancer is rapidly increasing and has the potential to inform the development of novel therapeutic approaches for this disease.

## 1 Molecular origins of breast cancer

Molecular heterogeneity is one of the archetypal features of breast cancer. This heterogeneity refers to the fact that breast tumors are composed of a mixture of cancer cells with different genetic and molecular characteristics. This diversity of features and origins *within a single tumor* can contribute to differences in tumor behavior, such as response to treatment and risk of recurrence. Research has identified several molecular subtypes of breast cancer, including estrogen receptor-positive, HER2-enriched, and triple-negative, each with its own unique set of genetic and molecular features. Additionally, within a given subtype, there can be further molecular heterogeneity, with different cancer cells possessing varying combinations of genetic and epigenetic alterations. This molecular heterogeneity can be driven by a variety of factors, such as inherited genetics, acquired mutations, and environmental exposures. Understanding the molecular diversity of breast cancer is important for developing personalized treatment approaches and for predicting patient-specific outcomes. However, the complexity of this heterogeneity presents a challenge for researchers studying the disease and for clinicians caring for breast cancer patients. Here we will present an overview of some of the molecular (mostly genomic and epigenomic) factors behind, and will discuss some of the already synergistic mechanisms giving rise to these complex pathophenotypes.

Aside (but not independently) from the genomic and epigenomic background, one additional source of complexity and heterogeneity in breast tumors is the influence of metabolic reprogramming in general, and hormone signaling in particular. Estrogen, for instance promotes cell proliferation, and on the other, it is oxidized to reactive products that damage DNA (1). Exposure to estrogen is linked to the menstrual lifetime, with a higher risk for women with early menarche and late menopause, but also to prolonged use of contraceptives and obesity. Before menopause, most of the estrogen in the body comes from the ovaries and a small percentage from fat tissue, but after menopause, the main source of estrogen is fat tissue, and the more there is, the greater the risk of breast cancer. In addition, being overweight causes a higher level of insulin in the blood, which has also been associated with breast cancer (2).

Two related factors that reduce the risk are the age of the first birth and breastfeeding. Experiments in mice show that pregnancy causes the differentiation of mammary lobules into secretory units, with lower proliferative activity, which would reduce the subset of cells susceptible to carcinogenesis. The risk reduction from breastfeeding is independent of childbirth and menopausal status, without a strong functional explanation, but several hypotheses, which include: interruption of ovulatory cycles, lower estrogen production and terminal tissue differentiation (1).

Despite the variation between countries and stages, breast cancer has a relatively good recovery rate compared to other types of cancer. It is estimated that up to 15% of patients develop distant metastases, which are mostly detected in bones, liver, lung and brain, with an association between the site of metastasis and the subtype of breast cancer (2, 3). In this regard, patterns have been identified that allow tumors to be grouped in different ways (4–6), which affect the prognosis and treatment of the disease, as we will discuss in the rest of this review.

### 1.1 Ductal and lobular origins of breast cancer

**Figure 1:**
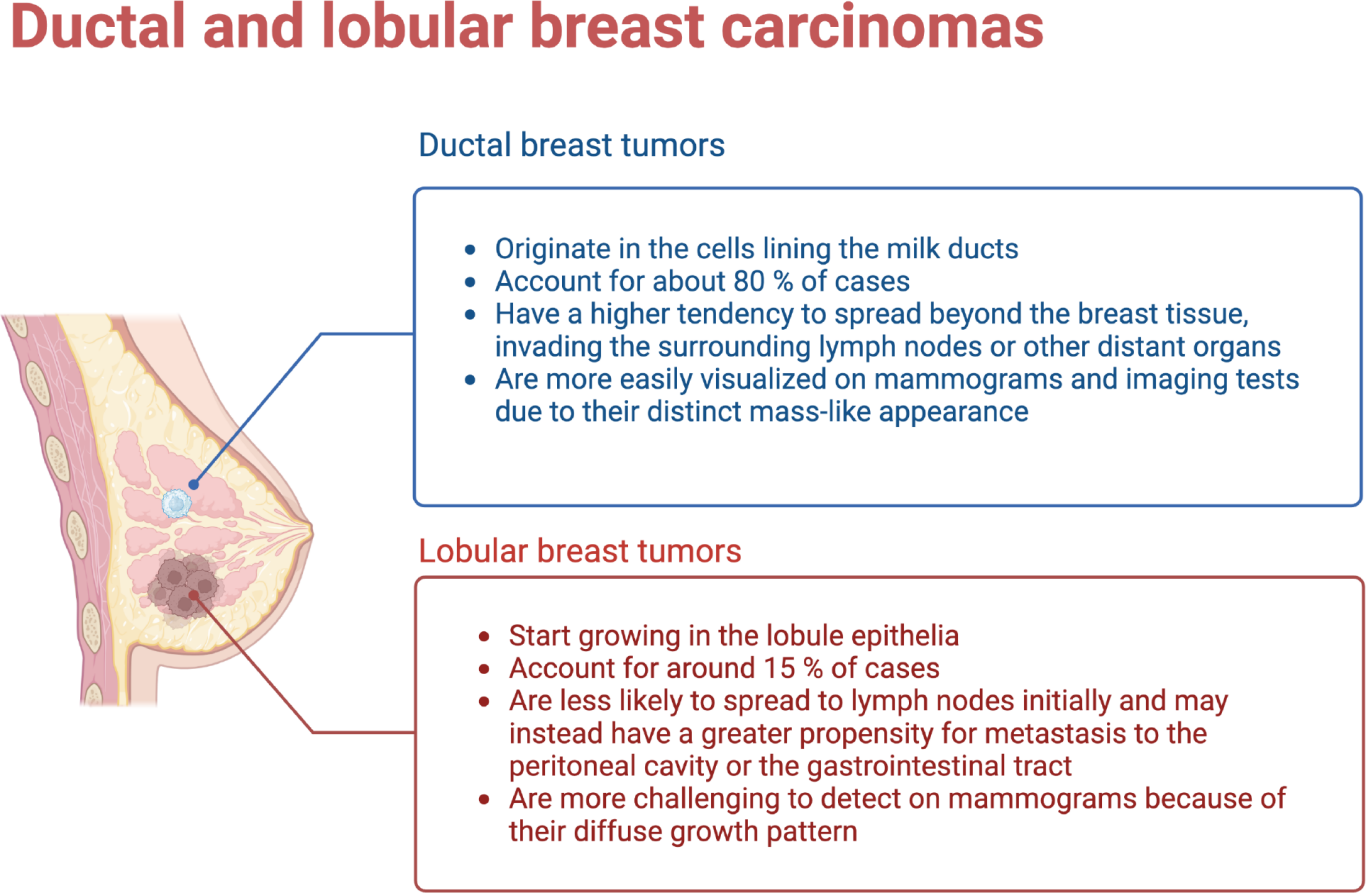
Ductal and Lobular breast tumors have different origins, development and outcomes. (Figure created using Biorender.com).

Breast cancer is a possess complex histological origins, as it is able to develop from different types of cells within the breast. Two common origins are the ductal and lobular tissues. Ductal carcinoma starts in the cells lining the milk ducts. These are thin tubes able to carry milk from the lobules to the nipple. Ductal carcinoma constitutes the most common type of breast cancer, accounting for about 80% of all cases. Typically appears as a lump in the breast that can spread to nearby lymph nodes if left untreated. Lobular carcinoma, on the other hand, originates in the lobules, the milk-producing glands within the breast. Lobular carcinomas are about 15% of breast cancer cases. Unlike ductal carcinoma, lobular tumors may not form a distinct lump. Instead, it often appear as a subtle thickening in the breast tissue. Lobular carcinomas are also able to spread to other parts of the body, including the opposite breast, ovaries, and abdomen (See **Figure 1**).

Ductal and lobular breast tumors show a number of genetic and molecular differences. These can be summarized as follows:

1. Genetic alterations: Ductal carcinoma characteristically display a higher frequency of genetic alterations in the tumor suppressor gene TP53. As is known, TP53 mutations are associated with more aggressive tumor behavior, hence poorer prognosis. Lobular tumors, in contrast often show alterations in the E-cadherin gene (CDH1) involved in cell adhesion. Mutations in CDH1 can lead to the loss of cell adhesion, a hallmark of lobular carcinoma.
2. Molecular subtypes: Breast cancer can be classified into different molecular subtypes based on gene expression patterns (see the next subsection). Ductal carcinomas are more commonly associated with the basal-like subtype, characterized by aggressive behavior and a higher risk of recurrence. Lobular carcinoma, are instead often classified within the luminal subtypes, which are typically less aggressive and associated with hormone receptor-positive tumors.
3. Hormone receptor status: Hormone receptor status, including estrogen receptor (ER) and progesterone receptor (PR) expression, differs between ductal and lobular breast tumors. Ductal carcinoma tends to have a higher frequency of hormone receptor-positive tumors, meaning they respond to hormonal therapies targeting these receptors. Lobular carcinoma is also hormone receptor-positive in many cases, but it has a higher tendency to have loss or reduced expression of hormone receptors compared to ductal carcinoma.
4. Metastatic patterns: Ductal and lobular carcinomas are also different in their patterns of metastasis. Ductal carcinoma often spreads to the lymph nodes and distant organs such as the lungs, liver, and bones. Lobular carcinoma instead has a higher propensity for multi-focal and multi-centric growth within the breast and has a greater tendency to metastasize to the peritoneal cavity, ovaries, and gastrointestinal tract.
5. Cellular morphology: Ductal carcinoma is characterized by the formation of irregular glandular structures, while lobular carcinoma often shows a characteristic single-file pattern, where the tumor cells infiltrate the breast tissue in a linear fashion without forming distinct masses.

We should note that individual breast tumors may indeed present a combination of features from both the ductal and lobular cellular origins. Moreover, as we shall see in the next subsection, advancements in genomic and molecular profiling techniques are allowing us to uncover additional subtypes and molecular features that contribute to further refine our understanding of breast cancer heterogeneity.

### 1.2 Breast cancer molecular subtypes

Most breast tumors affect the epithelium of the mammary glands, a mesh of branching ducts, which extend radially from the nipple and end in lobules (7). Therefore, histologically, they are carcinomas, which can be further classified as ductal or lobular and be invasive or presented in situ. The preservation of gene expression patterns indicates that invasive carcinomas often arise from *in situ* lesions (8). Less than 1% of breast tumors are sarcomas, which develop from the stroma of the glands, including blood vessels and myofibroblasts (2).

In connection with hormone and other signaling pathways, estrogen receptors (ER), progesterone receptors (PR), and human epidermal growth factor receptor 2 (HER2) have been used as immunohistochemical markers for clinical classification (5). The presence of estrogen receptors in up to 1% of tumor cells indicates a tumor that is (commonly) responsive to hormonal therapy (9), well-differentiated, and less aggressive. Tumors that are positive for HER2 may respond to treatment with monoclonal antibodies and kinase inhibitors, but the prognosis depends on the status of other receptors, among other issues. Tumors that are triple-positive usually have a good prognosis, while those with the ER-PR-HER2- phenotype are more aggressive and poorly differentiated. As a result, tumors without these receptors do not have targeted therapies and are generally of worst prognosis (10).

The relevance of the receptors has a biological reason, as estrogen stimulates the proliferation of cells with the receptor and induces the progesterone receptor - a mitogenic hormone - making PR+ tumors commonly also ER+ (10). On the other hand, the binding of the growth factor causes the heterodimerization of Her2 and the activation of its intracellular domain, which then participates in multiple transduction pathways, such as MAPK and PI3K (1).

To the previous sub-divisions, we must add the classification by means of gene expression, also called *molecular subtypes*. The classification by gene expression comes from the transcriptional patterns shared between different samples of the same tumor, which identify the intrinsic subtypes: luminal A, luminal B, Her2-enriched, and basal (4). Originally, a subtype (Normal-like) similar to normal tissue was also identified, but the possibility that it was instead formed due to contamination by adjacent normal tissue has kept the existence of this subtype in controversy (11).

Although different molecular classifiers have been used, such as Mammaprint and BluePrint, and even the subtypification with immunohistochemical markers of proliferation and the aforementioned receptors (12) has been approximated, the use of the PAM50 classifier (Prediction Analysis of Microarray 50), predominates in the databases and genomic studies. This is based on an array that measures the expression of the 50 genes that best separate the intrinsic subtypes (13) and provides highly predictive information on recurrence and neoadjuvant response (11).

**Figure 2:**
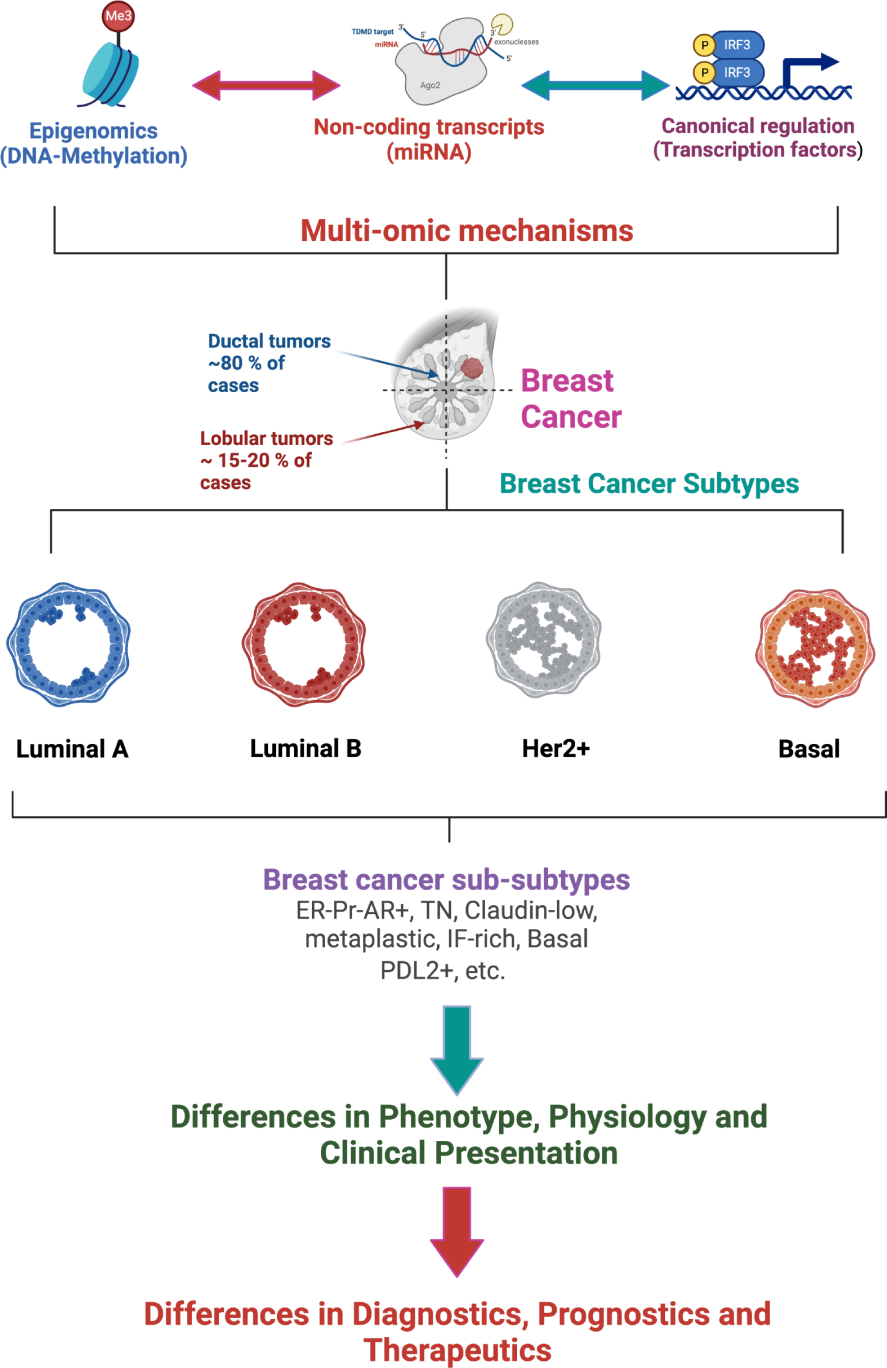
Multi-omic gene regulatory mechanisms influence breast cancer phenotypes affecting the classification, diagnostics, prognostics and therapeutics of breast tumors. (Figure created using Biorender.com).

**Figure 3:**
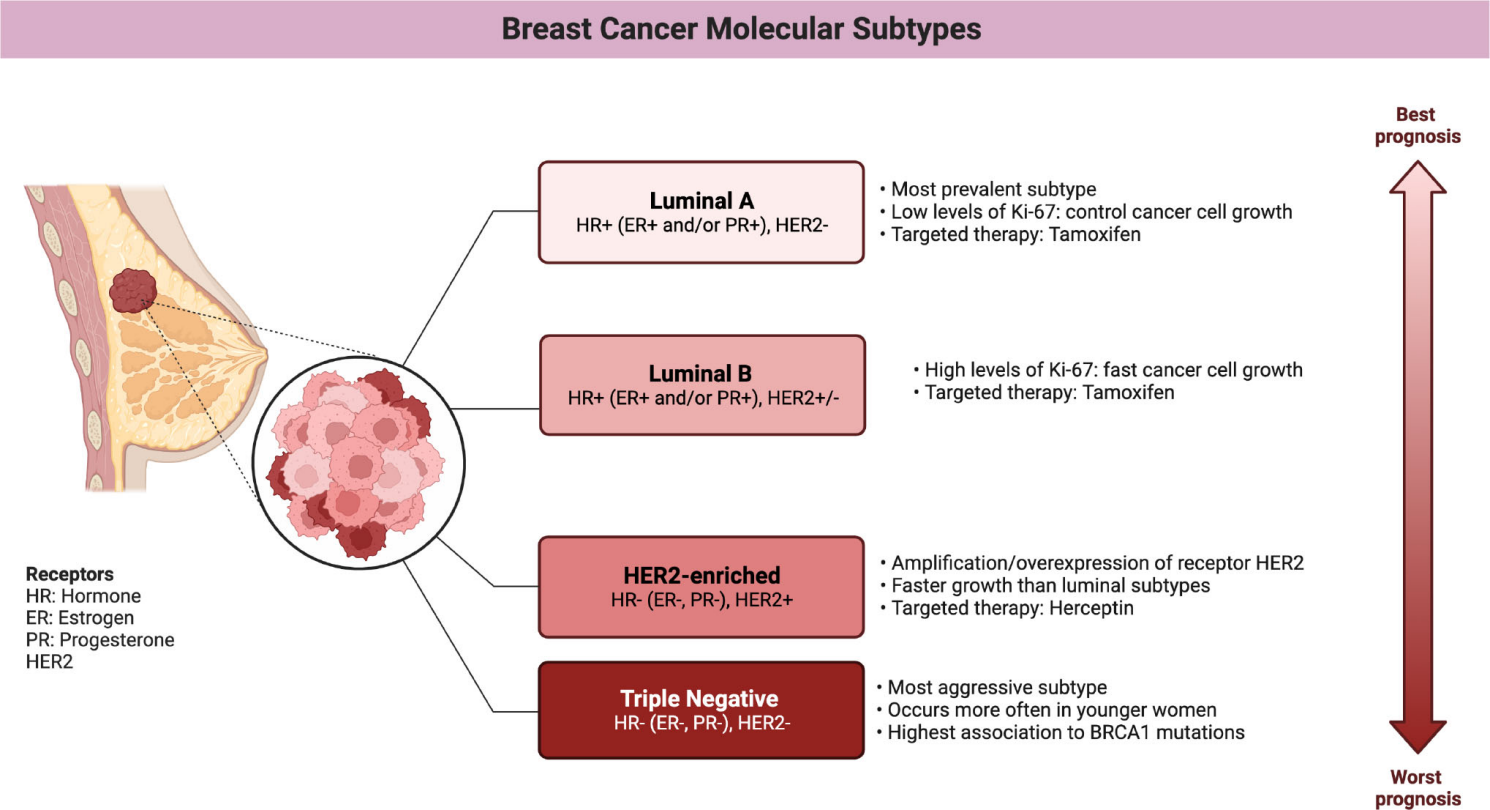
Main features of breast cancer molecular subtypes. (Figure created using Biorender.com).

The improvement of high-performance techniques enriched the description of breast cancer subtypes, allowing the transition from a grouping of transcriptional signatures to sub-subtypes with their own multi-omic characteristics (see **Figures 2**, **3**). In this way, the luminal subtypes can be clearly separated, as both are usually positive for hormonal receptors and negative for HER2; however, luminal B tumors have higher expression of genes associated with cell proliferation and lower expression of luminal tissue-related genes such as PR. A subset of luminal B tumors is characterized by hypermethylation of the Wnt pathway (5). Luminal A tumors have the lowest number of mutations but an increase in those affecting PIK3CA and MAP3K1 genes compared to the luminal B subtype. Interestingly, both subtypes have a good prognosis and high frequencies of around 30% of cases each, but luminal B tumors show greater chemosensitivity and the highest risk of recurrence in 10 years regardless of therapy. Therefore, this subtype has been proposed as the one to study, above others with a worse prognosis, to reduce mortality from breast cancer (13).

Tumors of the Her2-enriched (HER2E) subtype are characterized by the overexpression of HER2 and nearby genes such as GRB7, both at the transcriptional and protein levels, and by presenting the highest number of mutations in general and on the APOBEC3B cytidine deaminase gene in particular (13). HER2 overexpression has been associated with amplification of the long arm of chromosome 17, which nearly-always contains the receptor but whose extent varies. However, this subtype remains somehow controversial, as nearly half of the tumors with amplification are classified as luminal, mostly, or even basal (5).

Furthermore, differential expression studies between tumors with and without amplification identify few genes outside of chromosome 17, with modest changes. In contrast, when comparing HER2E tumors against non-HER2E tumors, the androgen receptor (AR) and different ER targets stand out, which could be explained by the metabolic and molecular redundancy between ER and AR. Adding the cooperation between HER2 and AR, as well as the inverse relationship between HER2 expression and ER/PR (10), it has been speculated that amplification could be a driver event that masks the hormonal nature of the subtype, as mostly apocrine (ER-PR-AR+) (14).

Basal tumors overexpress genes associated with cell proliferation and breast basal tissue, are characteristically hypomethylated, have the highest frequency of alterations on TP53 and are associated with the inactivation of BRCA1. When compared to different types of cancer, this subtype is molecularly more similar to squamous cell lung cancer than to luminal breast subtypes, while its pattern of mutations brings it closer to serous ovarian tumors. Although basal tumors would correspond to the triple negative (TN) phenotype of immunohistochemical markers, only 75% of TN tumors have the basal subtype expression pattern (5). This expression pattern is associated with aggressive tumors that present at early ages, with greater susceptibility in African ancestry populations and the worst prognosis at 5 years (13). The correspondence with the TN phenotype implies that there are no targeted treatments, however, the use of PARP inhibitors in tumors with BRCA1 mutations has recently been approved.

Molecularly, the basal subtype can be further divided, although there is still no consensus on how many and which those sub-subtypes would be, the subtypes *claudin low*, *metaplastic* and *interferon rich* have been consistently mentioned though (10). ATAC-seq studies identify categories similar to basal, mesenchymal and ligated to the luminal androgen receptor. Each category has its own mutations and clinical characteristics, of which the higher age of diagnosis for tumors ligated to the luminal androgen receptor and the activation without amplification of HER2 stand out. For their part, basal tumors are further separated into two groups, BL1 and BL2, according to the risk of progression. Those classified as BL2 are associated with an intact G1/S checkpoint, while those identified as BL1 lose copies of RB, which affects protein expression. Finally, mesenchymal tumors are characterized by a high percentage of mutations on epigenetic modifiers and DNA repair genes, as well as frequent deletion of beta-2-microglobulin, which suggests a reduced antigen presentation. Mesenchymal breast tumors also exhibit DNA hypomethylation, which coincides with greater chromatin accessibility over various enhancers (15).

The classification of basal tumors is particularly interesting because recently differences in the immune response of each subtype have been observed. For a long time, breast cancer was considered as poorly immunogenic given its relatively low mutational burden. However, a high survival rate has been observed among patients of the basal subtype with PDL2 overexpression, suggesting that a subgroup of breast cancer patients could indeed benefit from immune therapy (16).

When characterizing the basal subtype microenvironment, three groups were defined by 1) the inability to attract innate immune system cells, 2) chemotaxis followed by inactivation of innate immunity, and 3) increased immune inhibitory factors.

The phenotype of the first group has been explained by the amplification of MYC, which induces the expression of various chemokines and PDL1, as well as the inactivation of dendritic cells and macrophages, limiting the recruitment of adaptive cells. The second phenotype would be justified by the high infiltration of cancer-associated fibroblasts (CAF) (17), which correlates negatively with the infiltration of T cells (16) and depends on the immunomodulator TGFB; in addition to the inhibitory effect that the frequent mutation of the PI3K-AKT pathway would allow (17). Because of the characteristics of the third phenotype, this would be the subgroup of patients that could benefit more directly from immune therapy

After considering the enormous differences between the subtypes and sub-subtypes, it has been postulated that cells of different origin are involved (15). In principle, the luminal-basal division reflects the normal epithelium of the mammary gland, which is composed of a two-layer of luminal cells that produce milk and basal cells that expel the milk (8). Thus, the basal or myoepithelial layer is composed of contractile cells that express KRT14, TP63, ACTA2/SMA, MME/CD10, and THY1/CD90; while the luminal layer is formed by cells that are able to respond to hormones and express EpCAM, KRT8, KRT18, and MUC1 in addition to receptors. However, the luminal layer can be further divided into luminal cells and luminal progenitor cells. While luminal cells are clearly distinguished by their ER and PR receptor status, luminal progenitor cells almost completely lack these receptors and instead express KRT5/6, a marker of the basal layer in many types of epithelium. Different genetic expression characteristics and chromatin structure suggest that luminal progenitors may be actually intermediate cells between basal and luminal cells (7).

When examining the growth capabilities of each cell type, it was observed that all three types can generate colonies, but only about 0.1% of the basal fraction can produce two-layered structures similar to the mammary gland when injected into mice and, when appropriately stimulated, produce milk. Luminal progenitors only produce cells with luminal characteristics, with very short telomeres even when using samples from young women, and high levels of reactive oxygen species (7). In this way, according to the stem cell carcinogenesis model, poorly differentiated ER- tumors would arise from the most primitive cells - from the basal fraction -; Her2-enriched and luminal B tumors, which have been described as basoluminal, would come from an intermediate stem cell - the luminal progenitors -; finally, it is predicted that luminal A tumors would originate from the transformation of ER+ stem cells (8). Considering the limited division of luminal cells (7), the *clonal evolution carcinogenesis model* might be more appropriate for addressing the origin of luminal A tumors, as it proposes a population of genetically unstable cells that gain fitness by accumulating mutations and selection (8, 18). The origin of tumor cells is relevant because treatments typically eliminate proliferating cells, eliminating most of the tumor but often ignoring quiescent cells such as stem cells (8).

Beyond the origin of each subtype, it is clear that these are molecularly distinct entities and that these differences can affect their clinical behavior. Although this work delves into the transcriptional description, differences between subtypes can be observed at many other levels such as the rate of cis and trans interactions of the co-expression network (19) and the activation of metabolic pathways (20). Although it is not expected that intrinsic subtypes will replace immunohistochemical tests in the clinic soon, given the dependence on receptors for the assignment of treatments, nor should the tumor heterogeneity in these broad groups be oversimplified (14); molecular classification has been established as the unit of description of breast cancer and will be used throughout this work (See **Figure 2**).

## 2 Anomalous gene regulation in breast cancer

As data has been collected, the study of cancer has surpassed the reductionist approach that considered it simply *a disease of genes* (21). Thus, it has been considered a *disease of gene deregulation* (22, 23), *a disease of cellular processes* (24), a *disease of pathways* (25) and, when the origin of deregulation is considered, *a multi-scale disease*, where subcellular alterations affect the tissue, at the same time that the properties of the tissue -irrigation-, affect the phenotype and eventually the cellular genotype (26). In other words, a systems biology approach has been adopted, where interactions matter, whether they occur between genes or between scales. After all, it is not isolated genes that perform functions, but sets of proteins that have undergone regulated processes of transcription and translation and that need signals to enter into action or stop doing so.

The issue is that the regulation or deregulation of genes is already a multiscale problem, which at least involves regulatory sequences, transcription factors (TFs), histones, DNA methylation, non-coding RNAs, and chromatin conformation (27). The mentioned regulatory mechanisms can be organized into different categories, such as epigenetic, transcriptional, and post-transcriptional (as we will do in the rest of this review), but in reality they are interdependent and their simultaneous presence can be indeed identified in the same sample (28–30).

### 2.1 Epigenetic level: DNA methylation + transcriptomics

Epigenetic regulation involves modifications to chromatin that affect the binding of transcription factors. DNA methylation, specifically the addition of a methyl group to cytosine (5mC), is a well-studied mechanism in this process. Methylation primarily occurs in CpG dinucleotides, which are concentrated in CpG islands (CGIs) found in human genome promoters. Detection of DNA methylation can be done using sodium bisulfite treatment, sequencing, microarrays, methylation-sensitive restriction enzymes, or immunoprecipitation with antibodies against 5mC (22, 31–33). Microarrays, like the Illumina HumanMethylation450K BeadChip (HM450), have been widely used to characterize the methylome due to their cost-effectiveness and accuracy. Sequence-based methylation analysis such as the one carried out by sequencing bisulfite-converted DNA is a more comprehensive technique, able in principle to measure methylation at practically every cytosine in the genome (34). The method methods relies on bisulfite conversion of DNA to detect unmethylated cytosines. Bisulfite conversion changes unmethylated cytosines to uracil during library preparation. Converted bases are identified (following PCR) as thymine in the sequencing data, and sequencing reads are used to determine the fraction of methylated cytosines (35).

Methylation patterns generally correlate with CpG frequency, but CpG islands exhibit unique characteristics and play a role in transcriptional regulation (36–38). CGI promoters have distinct features and differ from other promoters in terms of transcription start regions, bidirectional transcription, and transcription factor binding sites (22, 39, 40).

DNA methylation plays a role in long-term genetic expression programming and cell type determination. After fertilization, the genome undergoes generalized de-methylation, followed by the establishment of *permanent* methylation patterns during embryogenesis (32, 41). *De novo* methylation occurs in early embryonic pluripotent cells, while maintenance methylation takes place during cell division, maintaining methylation patterns from the parental strand to the daughter strand. DNMT enzymes and S-adenosyl L-methionine are involved in DNA methylation, linking gene expression regulation to metabolism. To remove methylation, both passive and active mechanisms are proposed. The passive mechanism suggests that methylation is lost as cells divide, while the active mechanism involves TET enzymes. These mechanisms are associated with changes before implantation, with the maternal genome undergoing passive dilution of methylation and the paternal genome being influenced by Tet3. The gradual loss of DNA methylation observed with aging, particularly in monozygotic twins, may be attributed to the passive mechanism (42, 43).

The TET (Ten eleven translocation) protein family is a group of DNA hydroxylases responsible for oxidizing the methyl group of cytosine and its derivatives successively. The action of TET1, TET2 and TET3 catalyzes the conversion of 5-methylcytosine to 5-hydroxymethylcytosine (5hmC), which is converted to 5-formylcytosine (5fC), which in turn is oxidized to 5-carboxylcytosine (5caC). The 5fC and 5caC forms can be replaced by cytosines by the action of DNA glycosylase and base excision repair. The three derivatives are found simultaneously on the DNA, but cannot be specified by bisulfite sequencing, since 5hmC is read as 5mC, while 5fC and 5caC as cytosine. The identification of each form is relevant because, unlike 5mC, the derivatives do not allow the efficient binding of transcriptional regulators; but 5fC and 5caC favor the binding of proteins involved in DNA repair (44).

The binding of transcriptional regulators to methylated cytosines depends on proteins with MBD (methyl-CpG binding domain) domains, such as MeCP2, which also recruit histone deacetylases and methyltransferases and then reconfigure chromatin to its inactive form (41). Many TFs can bind to both methylated and unmethylated DNA, but with different affinities (45), such is the case of MYC, which binds to the CACGTG motif unless the central CpG has been methylated. Unlike MYC, methylation improves the binding of other transcription factors such as CEBPA and CEBPB (46).

The relationship between DNA methylation and transcription is complex and varied. While methylation of promoters generally inhibits transcription by blocking transcription factor binding, methylation of gene bodies can promote gene expression by facilitating transcriptional elongation (22, 31, 32, 41). However, there are diverse interactions between transcription and DNA methylation, including protection against methylation, promotion of methylation, and demethylation (46). Certain proteins, such as CFP1 and TET proteins, protect promoters from methylation by binding to non-methylated CpG sites. These proteins recruit methyltransferases or reverse *de novo* methylation. DNA-RNA loops resulting from active transcription have also been suggested to protect nearby promoters from methylation. On the other hand, transcriptionassociated proteins can promote DNA methylation by recruiting DNMTs. Examples include DNMT3B, MYC, and E2F6. The KRAB-ZNF family of transcription factors, characterized by an RH motif, can facilitate targeted methylation by interacting with DNMTs. Demethylation, on the other hand, involves the recruitment of TET proteins. Transcription factors like SPI1 and co-activators like PPARG can interact with TET proteins to induce demethylation or the conversion of 5mC to 5hmC in specific regions.

#### 2.1.1 Methylation and cancer

Considering the importance of DNA methylation on defining cell type through transcriptional regulation, it is understandable its alteration in syndromes and diseases. Prader-Willi, Angelman, Beckwith-Wiedemann and Silver-Russell syndromes have been mapped to chromosomal aberrations, but also to imprinting defects due to altered methylation of the involved genes: UPD, ICR2, and ICR1 (42). In cancer, levels of DNMTs expression have been reported similar to those observed in embryos, while TET enzymes mutation has been recurrently identified in different liquid tumors (47). The alterations in DNA methylation described in cancer are not limited to specific point mutations or epimutations –Epimutations are changes in the epigenome relative to consensus, equivalent to mutations (42), but reversible and more frequent (36), but include simultaneous hypermethylation and hypomethylation of multiple regions of the genome (41).

The hypermethylation of DNA in cancer affects 5-10% of CGI promoters - which are normally not methylated - and has been associated with the silencing of tumor suppressor genes (TSGs) (22), responsible, for example, for inducing apoptosis and cell arrest. In addition to epigenetic silencing, tumor suppressors often suffer disruptive mutations such as indels and stop codon substitutions in both alleles, as according to Knudson’s two-hit hypothesis, both copies of the gene must be inoperable for TSG inactivation (21). Promoter hypermethylation is usually the second impact of these genes and is thought to progress gradually, from the surrounding heterochromatin, to the transcription start site, subtly and heterogeneously reducing gene expression and favoring tumor plasticity (31). Thus, even the methylation coasts are differentially methylated in cancer. The number of affected CGIs also gradually increases as cell differentiation decreases (41).

Around half of the genes that cause familial forms of cancer can be found hypermethylated in sporadic tumors. In the case of breast cancer, 10-15% of women with sporadic tumors exhibit BRCA1 TSG hypermethylation, accompanied by an expression pattern consistent with hereditary tumors (31). Apart from tumor suppressors as such, hypermethylation causes harmful silencing of miRNAs and more complex deregulation, such as interference with ER-ERE binding (48) and loss of IGF2 imprinting. The expression of IGF2, involved in Beckwith-Wiedemann and Silver-Russell syndromes, is normally inhibited by the insulator H19, which prevents the action of a distal enhancer on the IGF2 promoter; however, in various types of cancer, H19 has been found to be hypermethylated, allowing the expression of the maternal IGF2 copy and causing excess growth factor. As with this, there are many examples of hypermethylation, to the point that filtering strategies are needed to identify their functional consequences (22).

Equivalently, hypomethylation causes the percentage of methylated CpG sites in the genome to drop from 80 to 60 or even 40% and progresses such that metastases have lower levels of methylation than primary tumors (49). The methylator phenotype identified in a subgroup of tumors is characterized by the coordinated methylation of a large number of CGIs, and has a low risk of metastasis and better survival rates. Taking advantage of these observations, agents have been found that reverse de-methylation, inhibiting the invasiveness and metastasis of breast cancer cell lines (41).

Unlike hypermethylation, hypomethylation does not occur in a focused manner on CGI promoters, but rather on a large scale, affecting repetitive elements that include transposons and oncogenes and mapping to late-replication regions associated with the nuclear lamina. Transcriptional activation of the repetitions predisposes the genome to recombination, as evidenced by the increase in the frequency of chromosomal alterations in cancer. Transposons are kept under control in basal tumors, due to compensation for the loss of methylation by trimethylation of lysine 27 of histone 3 (48). While hypomethylation promotes indels and translocations, methylation alone increases the susceptibility of cytokines to mutagenesis, because it increases the hydrolytic deamination rate, which, due to the methyl group, converts the base into thymidine instead of uracil, as corresponds to cytosines, preventing efficient repair of damage (22).

Although consistently an excess of variability in methylation levels has been found in breast cancer compared to normal tissue (49), specific patterns are known, at least for the basal, luminal B and Her2-enriched subtypes. The basal subtype is the most hypomethylated and, as expected, also has a high genomic instability. Among luminal B samples, a hypermethylated subgroup has been recognized, where the affected CpGs are linked to the Wnt pathway (5). On the Her2-enriched subtype, a bias towards hypermethylation - over hypomethylation - compared to normal tissue has been reported, which is associated with Her2 amplification and particularly affects Hox genes (50).

Although regulation by methylation acts locally on genes, coordinated methylation between distant loci can reflect the same transcriptional program. In that sense, it has been reported that more than half of the pairs of highly co-methylated genes - with Pearson correlation coefficients above 0.75 - in breast cancer are on different chromosomes and tend to participate in similar functions, with enrichment in the pathways of adult onset diabetes, hematopoietic lineage, long-term depression and interaction between receptors and the extracellular matrix (40). Saving the differences between studies, a pan-cancer analysis, which includes breast cancer, reports tissue variability, but identifies 4 groups of genes consistently co-methylated, two of which allow discriminating between cancer and normal tissue samples, despite containing only six cancer-associated genes: CSF2, GALR1, IRF4, PTPRT, SOX11y NRG1 (51). However, the levels of methylation and co-methylation do not necessarily imply a functional change in the cell, there are more regulatory mechanisms at play and it is estimated that only 15% of differentially methylated genes also exhibit a change in expression (50).

**Figure 4:**
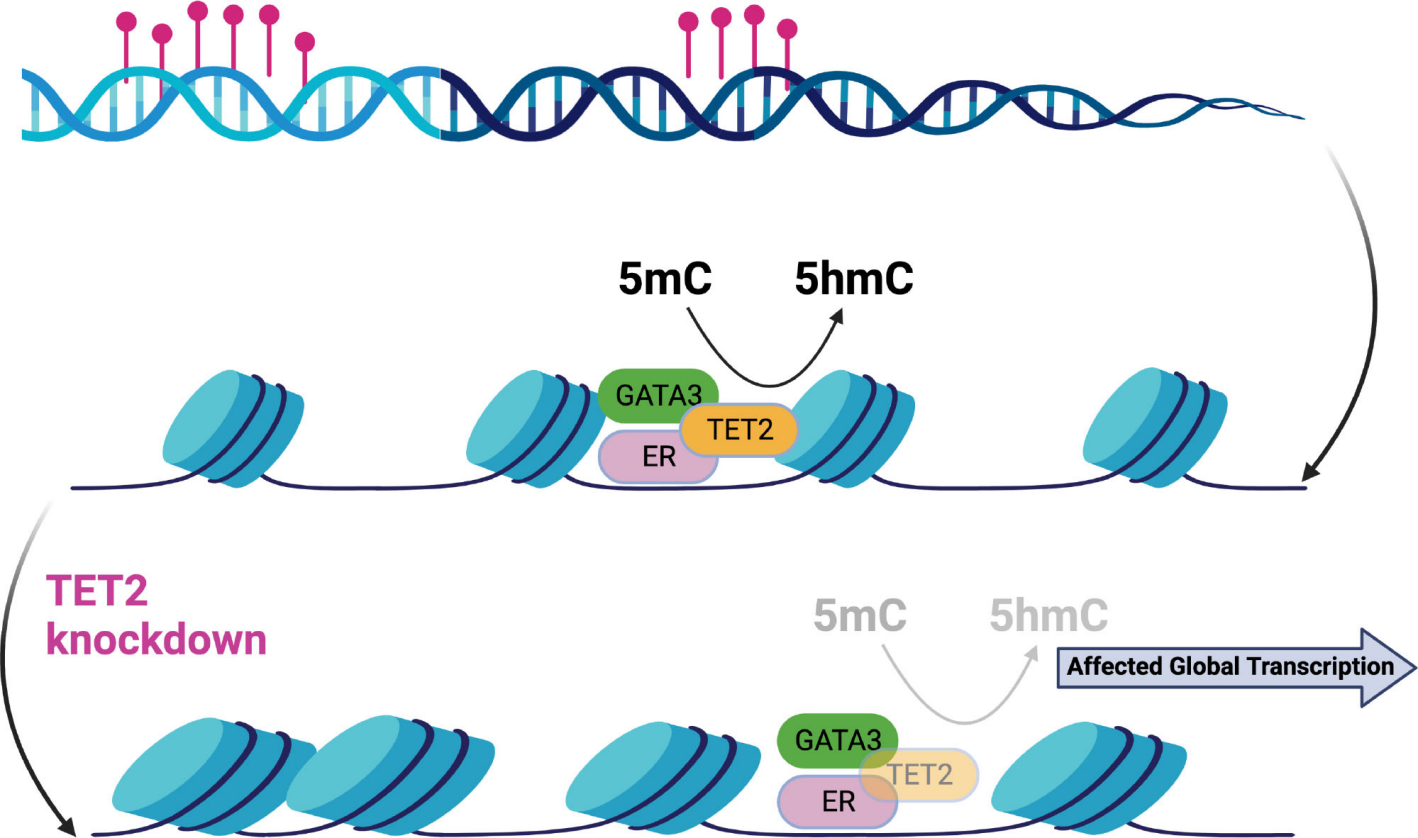
Epigenetic changes in the ER-complex may lead to global transcriptional deregulation in breast cancer [Figure created using Biorender.com, adapted from (52)].

Estrogen receptor-*α* (ER) drives tumor development in ER-positive (ER+) breast cancer. The transcription factor GATA3 has been closely linked to ER function. Epigenetic changes in GATA3 function may thus be relevant to breast cancer biology. It has been recently discussed how indirect changes in the activity of the transcription factor GATA3 by TET2 knockdown lead to epigenetic changes by significantly reducing 5-hydroxymethylcytosine (5hmC) levels without similar changes in methylated cytosine (5mC) (52). These changes are able to lead to global transcriptional deregulation (see **Figure 4**).

Other specific findings are opening new avenues of research in breast cancer biology. Such is the case of the recent discovery that overexpression of MAGI2-AS3 diminishes DNA methylation of MAGI2 in breast cancer cells (MCF-7) and thus would inhibit the Wnt/*β*-catenin pathway also diminishing cell proliferation and migration (53); the authors reported that MAGI2-AS3 may act as a cis-acting regulatory element down regulating the DNA methylation level of the MAGI2 promoter region.

In addition to providing information about the origin of tumors and potentially active genes, DNA methylation has gained clinical interest as a prognostic marker. DNA is a relatively resistant material that can be manipulated more easily than the RNA necessary to measure genetic expression (41) and that can be recovered from different bodily fluids depending on the type of cancer. As the tumor cells die, free DNA is released into the bloodstream, where it can be detected with high sensitivity (31). For example, from the levels of methylation in serum of women with metastatic breast cancer, a subgroup with higher disease-free survival could be distinguished, now recognizable by the methylation of SFN, hMLH1, HOXD13, PCDHGB7, RASSF1 and P16 (38). The hypermethylation of estrogen response elements is used to predict reduced response to endocrine therapy, with the methylation of PSAT1 as a specific indicator of response to tamoxifen. There are also numerous studies exploring the early detection of cancer using tests that measure DNA methylation. Both their sensitivity and specificity exceed those reported for mammography screening and are even higher for advanced stages (48).

The other potential usefulness of DNA methylation is in the treatment of cancer. The use of DNMT inhibitors as a sensitization strategy to other treatments is promising for breast cancer, although it has not yet been approved for routine clinical use. The DNMT inhibitors decitabine and 5-azacitidine are used in the management of hematological malignancies and can inhibit tumor growth in ER+ breast cancer models in combination with chemotherapy or immunotherapy. It is believed that these inhibitors activate the immune response by stopping the silencing of tumor antigens. What has been demonstrated is an increase in the expression of the immunomodulator PD-L1 in cell lines and xenografts treated with decitabine, which improves the recruitment of CD8+ cells and the effectiveness of immunotherapy. Additionally, a benefit has been reported in patients with BRCA1 methylation with the use of PARP inhibitors and it is believed that the epigenetic characterization of the response to CDK4/6 inhibitors could improve the management of patients with ER+ and metastatic breast cancer who receive this medication as first-line treatment, but do not always respond to treatment (48).

### 2.2 Transcriptional level: transcriptional factor analysis + transcriptomics

Transcription factors are a large family of proteins involved in the regulation of gene expression. They can be categorized into general factors involved in the transcription of most genes and sequence-specific factors that direct the spatial and temporal expression patterns of organisms. The ENCODE Factorbook database contains profiles of nearly 700 proteins related to transcription, including specific factors, cofactors, and members of the RNA polymerase II complex. Transcription factors can recruit RNA polymerase directly or rely on accessory factors for their function. Many eukaryotic TFs require co-activator or co-repressor complexes involved in chromatin remodeling. Some TFs interfere with the binding of other proteins (54–56). The HumanTFs database defines transcription factors as proteins that bind to DNA through a DNA binding domain (DBD) and regulate transcription. The database includes 1639 probable human transcription factors, with the C2H2-ZF and homeodomain DBDs being the most common. TFs often have multiple copies of a single DBD type and a combination of effector domains. The expression patterns of TFs largely depend on the DBD, with homeodomains showing tissue specificity. Transcription factors are often grouped based on the family of their DBD, which reflects the sequences they recognize. The largest families include C2H2-ZF, homeodomain, bHLH (basic helix-loop-helix), bZIP (basic leucine zipper), and NHR (nuclear hormone receptor), which were among the earliest described. This grouping by DBD family has its roots in homology and may have limited the identification of new factors, but it aligns with the evolutionary history of DBDs, which originated from a common ancestral set and underwent duplication and divergence (55, 57–59).

Transcription factors (TFs) can bind to specific sequences, called motifs, in the regulatory regions of target genes. These motifs are typically 6 to 20 base pairs long. Methods like ChIP-seq and HT-SELEX are used to identify binding sequences and determine motifs (56, 60). Weight matrices or hidden Markov models are then used to characterize the binding preferences of TFs, and databases like JASPAR store collections of motifs. However, the presence of a motif alone does not guarantee that a TF regulates a gene. Factors such as the accessibility of chromatin, DNA methylation state, nucleosome positioning, and interactions with other TFs also influence TF binding. Only the structural factor CTCF binds to almost 14,000 instances of its motif in the genome, while other TFs exhibit more complex binding patterns (61). TFs compete or interact with nucleosomes to access their motifs. The binding of TFs is associated with nucleosome repositioning, which is anti-correlated with DNA methylation levels. The absence of nucleosomes indicates a high and stable level of gene expression. Single-molecule tracking studies have shown that TFs transiently bind to DNA, and interactions between TFs can affect their diffusion dynamics (62).

Although transcription factors have been divided into activators and repressors, many TFs can recruit multiple cofactors with opposing effects, making it more appropriate to include the target and the condition under which a factor is operating. KRAB C2H2-ZF factors are repressors of transposable elements, by promoting their silencing (55); while HOXA5 functions as an activator of p53 in breast cancer cells (63, 64). Therefore, binding to the motif may be insufficient to determine the effect of the TF on the locus, and may simply reflect chromatin accessibility (54).

The binding of transcription factors (TFs) near the transcription start site can provide insights into gene expression levels. The binding of specific factors like E2F4 to non-methylated promoters explains a significant portion of gene expression variance, especially in CGI promoters. However, the predictive power decreases in promoters with low CpG density, suggesting the involvement of methylation in regulation. General factors contribute to a larger percentage of expression variance, and this percentage decreases when incorporating sequence-specific factors and histone modifications, indicating redundant regulatory mechanisms. Genes regulated post-transcriptionally, involved in cell cycle control and exhibiting tissue-specific expression differences, are particularly challenging to predict (54). In a study by Inoue and Harimoto, four expression patterns were identified among gene-TF pairs: no change, correlated expression, non-correlated expression due to constant TF levels, and lack of correlation due to variable genes. Correlated expression was associated with cell cycle and DNA replication genes, while various human diseases were linked to non-correlated expression. The lack of correlation revealed additional regulatory mechanisms. The third pattern, characterized by constant TF binding and gene degradation based regulation, was associated with genes whose expression is primarily determined by transcript degradation rather than synthesis. Disruption of degradation in such cases can have detrimental effects, as seen in the accumulation of the oncogene *β*-catenin (65).

#### 2.2.1 TFs in breast cancer

Transcription factors regulate a large number of biological processes and are essential for maintaining homeostasis, so it is not surprising that their alteration is associated with different diseases. In particular, TFs represent almost 20% of the identified oncogenes (47). However, transcription factors are not only affected by direct mutations, but the mutation and methylation of regulatory regions can also disrupt their binding and function (55).

In addition, there are a large number of transcriptional cascades triggered by the action of a few factors, which act as master transcriptional regulators. Master regulators are the genes that control the specification of a lineage either by direct or indirect regulation, whose altered expression can change cell fate (66). In breast cancer, AGTR2, ZNF132, and TFDP3 have been identified as master regulators linked to the distinctive features of cancer. Focusing on the signal transduction pathways, TSHZ2, HOXA2, MEIS2, HOXA3, HAND2, HOXA5, TBX18, PEG3, GLI2 and CLOCK were also identified, with the latter being the only positive regulator. Regulators in both sets show some redundancy in their targets, suggesting robust regulation. In the case of signal transduction, the *Hedgehog* pathway stands out for its relationship with morphogenesis and the self-renewal of stem cells (64, 67).

This relationship between cancer and cell differentiation and morphogenesis fits with the oncogenic theory of cancer, according to which, the aberrant expression of development genes allows the reprogramming of somatic cells to an immortal stem cell line of cancer cells, and then to a new cell identity (68). The epithelial-mesenchymal transition is a good example of this theory, as it depends on the same transcription factors - Snail, Slug, Twist and FoxD3 - during development as well as during cancer progression. Eventually, metastasis also resembles embryonic development of different structures, as it depends on the same morphogens: Wnt and Hedgehog ligands, bone morphogenetic proteins (BMPs), and fibroblast growth factors (FGFs) (47).

On the other hand, while the alteration of transcription factors or their expression modifies complete processes, the alteration of binding motifs also has an effect, perhaps more limited, by affecting only the relationship between the TF and a target gene, but equally problematic. When analyzing the accessibility of DNA in 23 different types of cancer, hundreds of non-coding and somatic mutations were found that affect the binding of transcription factors, suggesting a ubiquitous mechanism for manipulating genetic expression. The grouping of cancer types by DNA accessibility agrees with the grouping by multi-omic expression - expression of transcripts, microRNAs and proteins, in addition to DNA methylation and copy number - suggesting functional relevance (69). Accessible and specific regions of a group are hypomethylated compared to other clusters, while exhibiting enrichment of SNPs and cancer-associated TF motifs better represented in the cluster. Approximately 65% of these SNPs do not have the nearest gene as a putative target. When focusing on breast cancer, 36% of accessible regions were also accessible in other types of cancer, establishing a division between basal and non-basal tumors, and, as a result, a survival difference dependent on the accessibility of ESR1 motifs (6).

Of the 294 oncogenic TFs (70), the androgen and estrogen receptors, the BRCA1 and BRCA2 genes, MYC and GATA3 stand out for their association with breast cancer subtypes. The androgen receptor has been associated with the Her2-enriched subtype (14), although it also has clinical relevance, and is in fact more common in ER+ tumors (71). The estrogen receptor, on the other hand, is the marker par excellence of the luminal subtypes; while germline or somatic mutations of the breast cancer susceptibility genes and MYC activation are frequent in the basal subtype. Finally, the transcriptional factor GATA3 is particularly mutated in luminal tumors, where it is also often overexpressed (5).

Given the relevance of the estrogen receptor in the classification of breast cancer, it is worth delving into its functioning. In addition to its role as a transcriptional factor, ER is a member of the nuclear hormone receptor superfamily, encoded by the paralogs ESR1, on 6q25.1 and ESR2, on 14q22-24. The receptors that result from each gene, ERα and ERβ, respectively, have tissue-specific expression and differences in terms of structure and DNA binding, which nevertheless allow the formation of homodimers and heterodimers with a similar affinity for DNA. Steroid hormones diffuse through the plasma membrane and once the ligand binding domain of the receptor receives estrogen, a stable dimer is formed, capable of interacting with specific sequences through the DNA binding domain. The estrogen response elements (EREs) are palindromes of 5 base pairs separated by 3 bps, whose consensus sequence is GGTCAnnnTGACC. When the activated receptor binds to the ERE, it is believed that a pre-initiation complex for RNA polymerase is formed, through the inactivation or dissociation of co-repressors and the recruitment of co-activators, which favors cell proliferation (1).

In addition to the nuclear ER, there are receptors on the plasma membrane and in the mitochondria. On the membrane, the ER associates with lipid vesicles, interacts with growth factor receptors such as EGFR and HER2 and participates in non-genomic responses to estrogen, which range from the activation of kinases to the modulation of cellular migration, survival and proliferation. In the mitochondria, the presence of ERβ affects metabolism and anti-apoptotic signals (72).

The activity of the receptor changes with the nature of the ligand, phosphorylation and interaction with other TFs. The ER can promote transcription without hormone, either by interacting with the transcription factor Sp1 and its response elements or because extracellular growth factors cause phosphorylation and activation of the ER, crossing steroid hormone signaling pathways and receptors. The interaction with other TFs explains the activation of genes without ERE, while the interaction of ER with cyclin D1 allows the receptor to bind to EREs, also without estrogen and additively when there is hormone.

In addition, the function of the ER depends on the expression of the receptor, which is subject to regulation at multiple levels. The receptor promoter contains the motifs of different transcription factors such as Sp1, FoxA1 and Ezh2; in addition to several incomplete EREs. For its part, the six known isoforms of the messenger encode the same protein, but exhibit tissue-specific expression patterns and include different 5’UTRs, which seem to fold with more or less stability and could alter the efficiency of translation. On the other hand, the 3’UTR contains the seeds of 72 microRNAs, including miR-22, miR-206, miR-221 and miR-222, which are overexpressed in ER- tumors compared to ER+ and; the miR-17-92-miR-18a, miR-19b and miR-20b cluster, whose expression depends on ERα and cMYC, forming a negative feedback loop. Normally the 29 CpGs on ESR1 lack methylation, however extensive methylation has been documented in ER- cell lines (1).

The main alteration of the ER during the progression of breast cancer is in terms of its genetic expression. Although normal tissue only presents ER*α*, early ductal tumors have high levels of ER*α* and low levels of ER*β*, while in advanced stages both receptors are lost. On the contrary, lobular tumors begin with high levels of both receptors and end up losing ER*β* (73). Large disruptions and loss of heterozygosity rarely affect the receptor, so they cannot be used to explain ER- status. In other words, there are few documented mutations in primary tumors, which become frequent in metastatic lesions. For example, the Y537N mutant, which has been linked to bone metastasis and allows the constitutive activation of the TF, by abolishing the phosphorylation site. In addition, about 7% of tumors have mutations in the *enhancers* linked to ESR1 (7). Therefore, the alteration in breast cancer of the ER is more at the level of expression and has transcriptional effects.

Chromatin precipitation studies indicate between 5000 and 1000 EREs, which are reduced to approximately 1500 estrogen response genes (1). However, the effect of the TF is not solely local. Initially, it was described that ER*α*, FOXA1 and AP-2*γ* mediated the long-distance interaction between GREB1 and TFF1, but thanks to ChIA-PET studies, 689 chromatin loops formed by the interaction between distal and proximal EREs are now known. The loops are formed both intrachromosomally and interchromosomally and are believed to form subcompartments in the nuclear space (1, 74).

### 2.3 Postranscriptional level: microRNA expression + transcriptomics

MiRNAs are small, non-coding RNAs that regulate gene expression post-transcriptionally (36). They inhibit translation through base complementarity and can positively influence translation (75). MiRNAs are evolutionarily conserved, tissue-specific, and crucial for various cellular processes like proliferation and apoptosis (76–78). They can regulate a large portion of coding genes, impacting the cell’s gene expression profile (79). MiRNAs are abundant in somatic tissues and play a vital role in maintaining transcriptional network integrity (80). MiRNA production involves several steps: transcription of primary miRNAs (pri-miRNAs), recognition by the Drosha complex, formation of pre-miRNAs, export to the cytoplasm, processing by DICER, and transfer to the RISC complex (81, 82). Pri-miRNAs undergo cuts and modifications to become mature miRNAs, which play important roles in cellular processes (83). The production of mature miRNAs is efficient in healthy adult tissues (84).

MiRNA transcription can originate from their own promoters or coding gene promoters. They can be mono or polycistronic, with families sharing sequence similarity and functionality (75). For example, the miR-200 family is transcribed from different loci (81, 85). MiRNAs function as guides within the RISC complex, binding to messenger RNAs (mRNAs) in the 3’UTR region through miRNA response elements (MREs) (78). Binding leads to mRNA degradation mediated by argonaute proteins (77).

Predicting target messengers for miRNAs is challenging due to the size and low specificity of miRNAs. In addition to sequence information, conservation and thermodynamic stability play important roles. Various algorithms have been developed, including sequence-based and gene expression-based approaches that consider negative correlation or employ more complex methods (86). Databases like miRanda, TargetScan, and miRTarBase, which store predictions and validated cases, are valuable resources for miRNA target information (87).

#### 2.3.1 miRNAs in breast cancer

Counterintuitively given its pleiotropic role, many microRNAs are found in fragile regions of the genome and suffer from alterations in copy number (88), as seen with miR-125b, let-7g, miR-21, and 72.8% of miRNAs associated with breast cancer (23). While mutations on specific microRNAs have a limited effect, alterations to the miRNA production process affect the cell on an even wider scale, as they simultaneously alter multiple pleiotropic regulators. As a result, mutations in DROSHA and DICER are linked to low survival in patients with ovarian, lung, and breast cancer. Genetic expression alteration has been attributed to the regulators MYC and ADARB1 in the case of DROSHA, and miR-103/107 and let-7 in the case of DICER. Under-expression of DICER is associated with the basal subtype of breast cancer (77). Interestingly, there are miRNAs that are over-expressed when DROSHA or DICER are under-expressed, suggesting an alternative mechanism. The binding of KSRP to the RISC complex along with some pre-miRNAs, such as miR-21, posits this splicing protein, which is induced in response to DNA damage, as a possible part of that mechanism (82).

Other components of the microprocessor complex that are altered in cancer are DGCR8 and the helicases p68 and p72, which connect the microprocessor complex to p53. In the next step in miRNA production, inactivating mutations of XPO5 in tumors with microsatellite instability in colon, gastric, and endometrial tumors have been identified. The mutation of XPO5 increases the risk of breast cancer. The phosphorylation of XPO5 by MAPK/ERK in liver cancer has the same result as inactivating mutations, by preventing the export of pre-miRNAs to the cytoplasm. Outside the nucleus, factors associated with DICER, such as TARBP2 and AGO2, also exhibit alterations. Mutations in TARBP2 identified in carcinomas with microsatellite instability change the reading frame of the gene; while its under-expression is associated with melanomas and metastatic tumors of the breast and prostate. Over-expression of AGO2 has been reported in breast, gastric, and head and neck tumors (89).

Dependent on transcription, miRNAs are also modulated by DNA methylation and transcription factor binding. It is estimated that about 33% of the de-regulated miRNAs in cancer have alterations in DNA methylation (84). In cell lines without DNTM1 or DNTM3B, placentary miRNA expression is observed, normally silenced. In this regard, an important overlap has been reported between microRNAs marked by the Polycomb silencing complex in embryonic stem cells and those with CGI methylation in tumor cells (90). To mention a specific example, there is miR-205, whose sub-expression is associated with methylation of its promoter and resistance to treatment and epithelial-mesenchymal transformation (EMT) (75).

**Figure 5:**
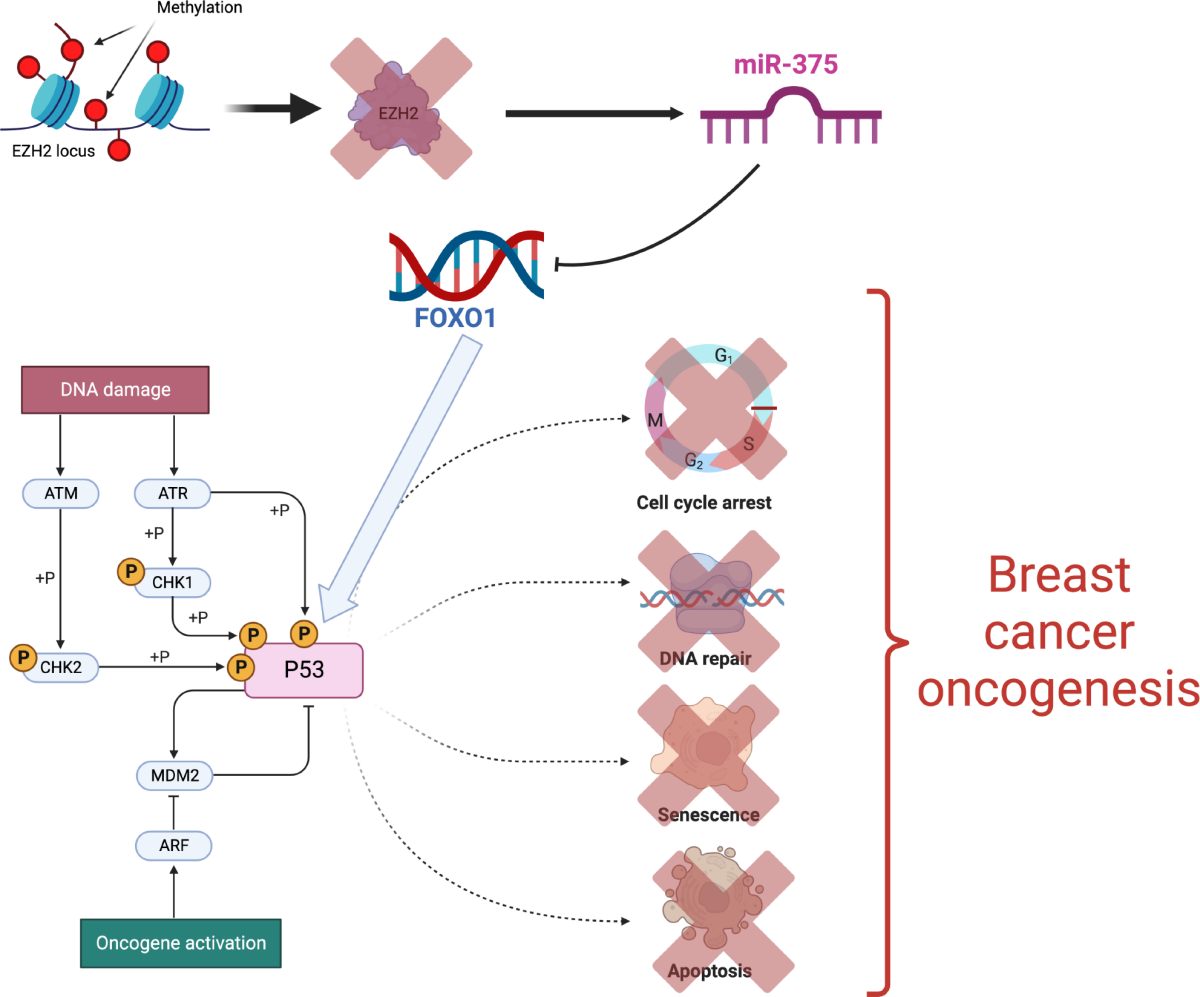
Epigenetic activation of the mir-375 oncogene [Figure created using Biorender.com, inspired from (91)].

Another recent example is upregulation of miR-375 *via* EZH2 methylation leading to FOXO1 inhibition. Inactivation of FOXO1 in turn promotes deregulated responses of the p53 pathway associated with breast cancer oncogenesis (91). Thus mir-375 has been recognized as a epigenetically regulated oncomir in breast cancer (**Figure 5**).

Examples of transcriptional regulation of microRNAs include regulation of MYC over miR23a and of NFkB over miR-29b (79). The case of miR-29 is interesting, because both regulators and effectors of the miRNA are known. MYC binding seems to be the initial step of silencing, and is followed by recruitment of histone modifiers. As part of the so-called *epi-miRNAs*, the miR-29 family inhibits DNMT3A, DNMT3B and DNMT1 (75, 79).

Finally, the tumor microenvironment can also alter miRNA levels, as observed in hypoxic breast tumors, where hypoxia inhibits oxygen-dependent histone demethylases KDM6A and KDM6B. As a result, the methylation - at the histone level - of the DICER promoter increases and its expression decreases, which also decreases the processing of miRNAs. The miR-200 family is one of the main ones affected by the sub-expression of DICER (82). By regulating the expression of transcription factors ZEB1 and ZEB2, which inhibit the transcription of epithelial genes such as E-cadherin; the loss of miR-200 favors the epithelial-mesenchymal transformation and is associated with metaplastic and aggressive breast tumors (76). In parallel to EMT, the loss of miR-200 releases the transcription factor ETS1 from the repression of the miRNA. ETS1 regulates the expression of angiogenic factors and, together with ELK1, triggers the methylation - at the DNA level - of the DROSHA promoter, further reducing miRNA levels, which has been associated with poorly differentiated tumors (76, 82).

On the other hand, it is common to find circulating miRNAs in fluids such as plasma and saliva. MicroRNAs in blood serum can even be used as prognostic biomarkers in breast, prostate, colon, ovarian and lung cancer. Specifically, the detection of mir-21, miR-92a, miR-10b, miR-125b, miR-155, miR-191, miR-382 and miR-30a would allow early identification of breast cancer (36). These miRNAs are protected from the action of RNAses thanks to their binding with lipoproteins and ribonucleoproteins or by their packaging in microvesicles (77). Once they are endocytosed, the regulation of translation in receptor cells is altered, involving microRNAs as signaling molecules. In this sense, it has been shown that cancer-associated fibroblasts secrete a different spectrum of miRNAs than normal fibroblasts, and these are not the only components of the microenvironment releasing microRNAs (78).

Even without considering circulating miRNAs, there is a clear difference between the profiles of normal breast tissue and tumors, with miR-10b, miR-125b, miR-145, miR-21 and miR-155 showing the most significant differences (88). In addition, miRNA expression profiles can distinguish between subtypes of breast cancer (5) and between cell subpopulations, with luminal progenitors being the cells most similar to basal tumors and mature luminal cells being the closest to luminal B subtype tumors. Luminal microRNAs regulate cell differentiation and development; while basal microRNAs regulate intracellular localization, organelle transport and biosynthesis, secretion and cell-cell interaction (92). Although the correspondence between intrinsic subtypes and miRNA profiles is noisy (5), the over-expression of miR-206 has been associated with ER- tumors and the under-expression of miR-125a/b with those enriched in Her2 (76).

MiR-206 inhibits the expression of ESR1; while its expression is favored by ER*α* and not by ER*β* or progesterone, suggesting a negative feedback loop. Other miRNAs that regulate ESR1 are miR-18a/b, miR-193b and miR-302c, whose expression, along with that of miR-206, causes cell cycle arrest and inhibits estrogen-dependent proliferation. In addition, miR-17-5p has the same effect, due to an indirect regulation of ER*α* through AIB1. The miRNA profile of breast cancer stem cells is also different, being enriched with miRNAs associated with self-renewal, such as let-7 and miR-34. Let-7 regulates oncogenes such as HRAS, HMGA2, MYC and caspase-3. In breast cancer over-expression of miR-34 causes cell cycle arrest and its under-expression increases invasive capacity (77).

miRNAs with a role in cancer can function as oncogenes or as tumor suppressors, depending on their targets. Tumor suppressor microRNAs inhibit the expression of genes that promote tumor development, so their sub-expression is harmful, as is the case with miR-200. OncomiRs, on the other hand, regulate tumor suppressors and it is their over-expression that is harmful, as is the case with miR-21, which regulates promoters of apoptosis and cell migration (77). In addition, a subcategory of oncomiRs could be defined with miRNAs exclusively pro-metastatic, such as miR10b and the miR-373/520c family. It has been reported that miR-10b is over-expressed only in metastatic breast cancer cells and not in the primary tumor; miR-10b inhibits the transcriptional factor HOXD10 and, in doing so, triggers a cascade of changes that ultimately lead to pro-metastatic RHOC expression, cell migration, and invasion (76).

However, the role of a miRNA could depend on the cellular context, as miRNA-mRNA regulatory interactions may not necessarily exist in all types of cancer (86). In a pan-cancer, computational study of miRNAs that direct genetic expression, it was observed that miRNA-gene interactions are not conserved, even though there are 22 miRNAs that do function as *drivers* in different types of cancer. Except for miR-5001 in colorectal cancer and miR-2276 in endometrial cancer, in this study all miRNAs are classified as tumor suppressors and the let-7 family functions as a TSG and as an oncomiR at the same time (93).

Despite the fact that each miRNA can regulate hundreds of genes, miRNAs have been proposed as possible means to regulate cancer genes, either by introducing oligonucleotides similar to miRNA to restore miRNA expression and suppress oncogenes, or by introducing antagonists to inhibit the miRNA of interest. An example of antagonists or antagomiRs are miRNA sponges, synthetic messengers with multiple binding sites for a specific miRNA, which then capture it, preventing it from inhibiting TSGs. There are formulations of miRNA-like oligonucleotides, miRNA sponges, anti-miRNA oligonucleotides, and small molecules that are being studied in cancer models. For breast cancer, at least an antagomiR-10b and a miR-195-like oligo have been tested. The antagonist inhibits lung metastasis, but not the growth of the primary tumor in mice; whereas the miR-195-like oligo increases sensitivity to treatment and inhibits Raf1 and Bcl2 translation in cell lines (77, 78).

## 3 Single cell breast cancer multi-omics

In recent times, multi-omic approaches have been further advanced with the advent of single cell sequencing techniques that have allowed for the integration of transcriptome data, as well as, other omics such as ATAC-seq (Assay for Transposase-Accessible Chromatin using sequencing) (94) to achieve a deeper understanding of molecular profiles and its functions at the level of a single cell or cell-type. These already outstanding methods are being further advanced by the integration of spatial multi-omics (95). The goal of *spatial* approaches is to be able to assign cell types (as identified by the mRNA and other omic sequencing readouts) to their locations in the histological sections of a given sample tissue. Spatial omics allow, for instance, to uncover cellular heterogeneity in tissues, tumors, immune cells as well as determine the subcellular distribution of biomolecules in diverse phenotypes.

Single cell and spatial multi-omics are thus becoming relevant tools and methods to analyze cancer biology from its basic principles (e.g. oncogenesis) (96) to the way these tumors evolve and their related outcomes by allowing to account for issues such as how dynamic processes and clonal selection manifest in cellular states, epigenetic profiles, spatial distributions and interactions with the microenvironment (97, 98).

Single cell studies in breast tumors, although quite recent, are starting to render fruits in the understanding of breast cancer heterogeneity as exemplified by the recent discovery of two lipid-associated macrophage states LAM1 and LAM2 (99) that are being established as biomarkers distinct clinical outcomes in several breast cancer datasets (100). Single cell multi-omics is also being used to develop strategies for clinical trial evaluation and drug discovery (101).

## 4 Applications of concerted multi-omic regulation analysis in breast cancer

The concurrent activity of several biological processes as measured by diverse omic technologies is paving the way towards advancing, both our knowledge about breast tumor biology and our therapeutic approaches. A number of these advances have ben summarized recently by Mehmood and collaborators (102). These authors have described the power of multi-omics to face the challenges of multidrug resistance (MDR) and relapse in breast carcinoma treatment. They emphasize the importance of elucidating multi-omic mechanisms to design therapies able to overcome drug resistance. Since breast carcinoma treatment decisions rely not only on prognosis factors but also on the assessment of pathological and clinical factors, the integration of data from multiple factors through a multiomics approach can provide valuable insights for therapeutic decisions. Along the same lines Ektefaie et al. (103), describe the development of weakly supervised deep learning models for analyzing multiomics from breast cancer biopsy samples. These automated models developed for tumor detection and pathology subtype classification demonstrated high accuracy and were validated in independent cohorts.

Regarding the interplay of epigenomic and genomic features, it has been discussed (104) that the CT83 gene is frequently activated in triple negative breast carcinomas (TNBC) and several other cancers, while it remains silenced in non-TNBC, normal nontestis tissues, and blood cells. A significant correlation was found between hypomethylation on chromosome X and the abnormal activation of CT83 in breast cancer. Furthermore, the activated CT83 was associated with unfavorable overall survival in breast cancer and worse outcomes in other cancers. The authors argue that abnormal activation of CT83 is likely oncogenic by triggering cell cycle signaling. Also in the context of TNBC multi-omic studies (17) combined with immune profiling have revealed a classification of the microenvironment phenotypes in triple-negative breast cancer (TNBC) into three distinct clusters. Cluster 1, known as the *immunedesert* cluster, exhibits low infiltration of microenvironmental cells. Cluster 2, referred to as the *innate immune-inactivated* cluster, demonstrates the presence of resting innate immune cells and nonimmune stromal cells infiltration. Lastly, cluster 3, the *immune-inflamed* cluster, shows abundant infiltration of both adaptive and innate immune cells. The clustering results were validated internally using pathologic sections and externally using The Cancer Genome Atlas and METABRIC as independent cohorts. These microenvironment clusters also displayed significant prognostic efficacy. The authors describe potential immune escape mechanisms associated with each cluster. Cluster 1 is characterized by an inability to attract immune cells, with low immune infiltration correlated with MYC amplification. In cluster 2, chemotaxis but innate immune inactivation and low tumor antigen burden potentially contribute to immune escape, with mutations in the PI3K-AKT pathway possibly associated with this effect. Lastly, cluster 3 is distinguished by high expression of immune checkpoint molecules. A similar approach to classification was made by Coria-Rodriguez and coworkers (105) to infer epigenomic signatures to define TNBC classes with differential response to therapy with drug repurposing goals in mind.

Tumor metabolic reprogramming has been studied with a multi-omic strategy by Iqbal and his group of collaborators (106) to show that there are antagonistic roles of CBX2 and CBX7 in metabolic reprogramming of breast cancer. They identified significant roles of CBX2 and CBX7 in positive and negative regulation of glucose metabolism and provided functional evidence for the mTOR complex 1 signaling in mediating competing effects of CBX2 and CBX7 on breast cancer metabolism. Disease-specific survival and drug sensitivity analysis revealed that CBX2 and CBX7 predicted patient outcome and sensitivity to FDA approved/investigational drugs.

Muli-omic analysis have also provided relevant clues, for instance on the role of lipid metabolism for the development and outcomes on early breast carcinomas (107). Concurrent ultrahigh-performance liquid chromatography-mass spectrometry experiments along with transcriptomics, and genomics data led to the identification of 18 oxylipins, metabolites of omega-3 or omega-6 polyunsaturated fatty acids, that were differentially expressed in breast tumors versus healthy sample tissues, including anandamide, prostaglandins and hydroxydocosahexaenoic acids. The authors hypothesize that oxylipin signatures reflect the organism’s level of response to the disease and may become markers of malignancy.

Tumor survival and drug-response predictions have been discussed at the light of breast cancer multi-omics (108) aimed on quantifying survival and drug response. The framework utilizes Neighborhood Component Analysis (NCA) for feature selection from multi-omics datasets obtained from The Cancer Genome Atlas (TCGA) and Genomics of Drug Sensitivity in Cancer (GDSC) databases. A Neural network framework, fed with the NCA selected features, is used to develop prediction models for survival and drug response in breast cancer patients. The results demonstrate a strong linear relationship between predicted and actual IC50 values outperforming previous approaches and highlighting the importance of multi-omics data integration.

The improved knowledge provided by multi-omic studies is also impacting on novel treatment designs such as immunotherapy, as has been recently summarized in the review work by Leung, et al. (109); a well as clues helping to advance druggable targets and autophagic modulators such as SF3B3 and SIRT3, that may improve the treatment of invasive breast carcinomas (110); and on exploiting the therapeutic and diagnostic value of IMMT in breast cancer as well as its immunological role (111). Immune infiltrate activity in breast tumors has been also further clarified by multi-omics as exemplified by the work of Tian and collaborators (112) about the relationship plasmacytoid dendritic cells and breast cancer.

Multi-omic strategies have also allowed to discern particular sets of biomolecular interactions relevant to certain aspects of breast cancer biology. Some of these interactions are indeed becoming interesting clues towards targeted therapy. Such is the case of the mechanisms by which the mitochondrial protease ClpP is activated by drugs that are able to breakdown essential mitochondrial pathways in triple-negative breast cancer (113). Similarly, the role of heat shock proteins (which may be either acting as oncogenes and onco-suppressor genes) has been recently discussed at the light of multi-omic analysis (114). Discerning the mechanisms of novel therapeutic drugs such as signaling inhibitors is crucial on our advance towards precision therapeutics of breast cancer. In this regard, Marczyk and collaborators (115) have studied the effects of navitoclax, a BCL2 family inhibitor, on the transcriptome, methylome, chromatin structure, and copy number variations of MDA-MB-231 triplenegative breast cancer (TNBC) cells. They were able to derive an 18-gene navitoclax resistance signature. Other pharmacological resistance mechanisms have been further elucidated. For instance, methylation events leading to HSD17B4 silencing have been identified as part of a predictive and response marker of HER2-positive breast cancer to HER2-directed therapy (116).

Breast cancer multi-omic integration tools have been recently developing at a fast pace. In order to better exploit the available and upcoming resources, researchers at the Chinese Academy of Sciences implemented MOBCdb a database integrating multi-omics data on breast cancer (117). MOBCdb is a user-friendly and readily available database that combines genomic, transcriptomic, epigenomic, clinical, and drug response information from various subtypes of breast cancer. It offers a convenient platform for users to access simple nucleotide variations (SNV), gene expression, microRNA expression, DNA methylation, and specific drug response data through different search methods. Additionally, the genome-wide browser and navigation feature in MOBCdb enable simultaneous visualization of multi-omics data from multiple samples.

## 5 Conclusions

We have discussed how various types of gene regulatory phenomena in breast cancer arise from several omics data, such as gene and non-coding RNA transcriptomics, methylation and transcription factor activity, as reported in the recent literature. We have also discussed how this knowledge can be integrated to provide a more comprehensive understanding of gene regulation in breast cancer, highlighting the importance of considering the spatial and temporal context in which gene regulation occurs, as well as the role of regulatory elements such as non-coding RNA and epigenetic modifications. In this regard, recent advances in single cell approaches to breast cancer multi-omics have been also presented. Some applications to tumor sub-classification, prognosis and survival analysis, drug repurposing and personalized therapeutic designs were introduced.

For concreteness, other potentially relevant aspects of the complex regulatory patterns in breast cancer have been left out for future discussion. Such is the case of the role played by copy number variants, long non-coding RNAs and the multi-scale three dimensional structure of nuclear chromatin. However, by considering the levels discussed in this review article, we have tried to unveil the potential of multi-omic approaches to improve our understanding of the complex molecular processes underlying breast cancer that may hopefully help us identify new therapeutic targets.

## Author contributions

Both authors performed research. SO drafted the first version of the manuscript, EH-L edited and rewrote the manuscript. All authors contributed to the article and approved the submitted version.
